# The relationship of sleep problems between eight-year-old South African children and their mothers

**DOI:** 10.7189/jogh.15.04122

**Published:** 2025-09-26

**Authors:** Mary Jane Rotheram-Borus, Joan Christodoulou, Lauren D Asarnow, Peter P Norwood, Matt Yalch, Steyn L Vogel, Mark Tomlinson

**Affiliations:** 1 Semel Institute Center for Community Health, University of California, Los Angeles, California, USA; 2 Department of Psychology, Palo Alto University, Palo Alto, California, USA; 3 Department of Psychiatry & Behavioral Science, University of California, San Francisco, California, USA; 4 Department of Global Health, Institute for Life Course Health Research, Stellenbosch University, South Africa; 5 School of Nursing and Midwifery, Queens University, Belfast, UK

## Abstract

**Background:**

Sleep problems are common among children and mothers. However, little is understood about sleep behaviours in low- and middle-income countries. Here we examine sleep behaviours and predictors among black, low-income, South African mothers and their eight-year-old children over time.

**Methods:**

We administered standardised measures of sleep behaviours at eight years post-birth to a population cohort of mothers and children in 24 neighbourhoods of Cape Town, South Africa, in 2009–10. We assessed mothers and children six times over eight years with 84% retention since pregnancy. While 71% remained in Cape Town, about 29% of households moved to the profoundly rural Eastern Cape of South Africa. Mothers completed the Pittsburgh Sleep Quality Index and rated their children on the Children’s Sleep Habits Questionnaire.

**Results:**

Among mothers, 29.5% reported sleep difficulties based on their global sleep score, with a mean sleep quality score of 3.72 (standard deviation (SD) = 2.6). Children's sleep scores were 64.4 (SD = 4.0), with subscale scores on sleep difficulties higher than documented in high-income countries (HICS). There was a relatively low inverse relationship between the quality of maternal and child sleep (*r* = −0.201; 95% confidence interval (CI) = −0.264, −0.136), which resulted in an R^2^ value of 0.041 in the simple linear regression model. Problematic maternal sleep was associated with living in the rural Eastern Cape (*P* = 0.034), experiencing intimate partner violence (*P* = 0.052), and a higher score on the Edinburgh Postnatal Depression Scale (*P* < 0.001), but not alcohol use. Children's sleep difficulties decreased by 0.191 points (95% CI = −0.229, −0.152) with a one-unit increase in aggressive behaviour and, similarly, cognitive scores decreased by 0.035 points (95% CI = −0.063, −0.006). Household resources, such as formal *vs.* informal housing, income, and having water on the premises, were unrelated to sleep difficulties.

**Conclusions:**

Counter to hypotheses, a small, inverse relationship existed between mothers' and children's sleep behaviours. Alcohol use, HIV status, and socioeconomic markers were unrelated to sleep, but intimate partner violence and depressive symptoms affect sleep negatively, similar to HICS.

Sleep problems are common among children. Up to 50% of middle-childhood-aged children experience a sleep problem, and about 4% will be diagnosed with a sleep disorder, such as insomnia or obstructive sleep apnoea [1,2]. Common sleep problems include difficulty falling asleep, middle-of-the-night awakenings, and snoring [2]. The consequences of poor sleep among children are well documented in high-income countries (HICs), including the immediate challenges of excessive daytime sleepiness and poor daytime functioning, as well as long-term associations with poor physical, cognitive, and behavioural health [3]. Sleep problems among children in low- and middle-income countries (LMICs) are poorly understood, so we focussed on this group.

Sleep problems also occur frequently among mothers, often beginning in pregnancy [4] and continuing through their children’s early [5,6] and middle childhood years [7]. More than half (60%) of pregnant women experience sleep problems, with about 40% during the post-partum period in Ethiopia [5] and at similar rates in Norway [7]. Again, almost all the evidence on sleep issues is from HICs [8]. Sleep patterns in LMICs are understood far less, especially in Africa.

Mothers’ and their children’s sleep appear to be significantly correlated in HICs [9–11]. A recent systematic review suggests a bidirectional relationship between mother and child sleep, including sleep quality, insomnia symptoms, and objectively measured sleep duration and efficiency [3]. Yet, the relationship between maternal-child sleep and the predictors of poor sleep are rarely investigated in LMICs. Based on data from HICs, we expect a relatively strong relationship between maternal and child sleep; the better a mother’s sleep, the better the child will sleep and vice versa.

However, the sleeping conditions are significantly different in HICs and LMICs. Sleep studies consistently find sleep influenced by the climate (*i.e.* temperature and humidity), synthetic light, size and placement of the beds, cultural norms, and households’ economic resources [9–12]. Households in LMICs often live in crowded areas in informal dwellings or shacks, typically without access to water or a flush toilet on site [12–14]. It is common for large extended families to live in one household. Climate control during sleep is rare; light from streetlamps persists all night, and adults can watch TV late into the night next to children’s sleeping spots [13,14]. In an informal dwelling, there is likely to be only one or two beds, a factor that may easily be linked to poor sleep [12].

Co-sleeping, a situation in which the mother and child sleep in the same bed, is common in some African LMICs [9–15]. While the data were reviewed 20 years ago, 10–23% of 5–11-year-old children were co-sleeping [13–15]. Most of the research on co-sleeping focusses on infancy while mothers are breastfeeding [8]. However, in many LMICs, co-sleeping continues for various reasons – a lack of resources reduces the availability of beds and separate spaces for parents and children to sleep, and local cultural norms endorse far more sharing at older ages. These conditions are likely to have more proximal influences on children’s sleep in informal dwellings compared to more stable, formal housing settings [11].

In addition to the physical sleep setting, family characteristics, such as low socioeconomic status and household chaos, decrease the quality and duration of sleep [9–15]. Both maternal health and habits of daily living are linked to sleep. In this sample, about 26% of children are born to mothers living with HIV (MLH), and an additional 25% of mothers acquire HIV in the next eight years. Both interpersonal violence and depressed mood are common; 34% of South African mothers report depressed mood during and after pregnancy, and 20% are physically abused [15,16]. MLH are more likely to experience depressed moods, potentially affecting mothers’ sleep [15]. In both HICs and LMICs, maternal depression has been associated with poor sleep [15,16]. Mothers are more likely to experience depressive symptoms if their infants have poor sleep [17]. About 25% of pregnant women abuse alcohol, and alcohol use is inversely associated with poor sleep [18,19]. Similar relationships exist between sleep and interpersonal violence [20] and sleep and employment [21]. These relationships, however, are typically assessed in HICs. We hypothesise that similar relationships will occur in the townships in Cape Town, South Africa.

Poor sleep is consistently associated with negative consequences on children’s growth, behaviour, and cognitive functioning, regardless of income status [3,22]. Yet, most children do not sleep the recommended 9–11 hours per night [23]. For children in middle childhood, sleep problems have been associated with excessive weight gain in both HICs and LMICs [22]. Furthermore, poor sleep has been associated with unintentional injuries among children in China [24]. Studies also suggest that chronic and occasional sleep problems can significantly affect children’s daytime functioning, including poor emotional regulation, short-term and working memory, and attention [25].

In our study, we assess the quality of children’s sleep at the age of eight, the relationship of children’s sleep to that of their mothers, and how children’s sleep is related to their physical, behavioural, and cognitive health. We obtained the data for these analyses from a population cohort studied longitudinally at six time points from pregnancy to eight years of age. Initially examining the impact of home visiting, all benefits of this perinatal intervention dissipated in early childhood [26–32]. We also aim to assess maternal and child sleep among 98% of children born in 24 Cape Town areas in 2009–10, a population cohort of all births in defined geographical settings, 84% of whom were retained and reassessed six points over the next eight years. Yet, as these children grew, 29% of the children were moved to deeply rural communities in the Eastern Cape of South Africa, and half of the children in Cape Town relocated from informal shacks to formal housing. These households’ movements allow us to observe sleep in varying circumstances – from overcrowded shacks in low-income urban and peri-urban areas to huts with low density in farming communities in rural areas. Thus, we obtained a representative sample of mothers and children eight years post-birth, whose health and life circumstances have been monitored over time.

## METHODS

### Participants

We identified household clusters (n = 24) of 450–600 households and matched them based on the housing type, presence of electricity, water, sanitation, size and density, the number of illegal, informal alcohol bars, childcare resources, distance to clinics, and the length of residence. Only 2% of infants born in these 24 neighbourhoods were not enrolled in the study, resulting in a sample of 1238 mother-child pairs. By birth, 69 mothers had miscarried, or children died in childbirth; another 34 children died within the next eight years; 24 mothers also died over eight years. Our exclusion criteria for this study’s analyses included maternal/child death (n = 127) and twin and triplet births (based on different developmental trajectories) (n = 13). Interviewers at a research office located in Khayelitsha conducted follow-up assessments at two weeks post-birth (92%), 0.5 years (87%), 1.5 (91%), 3 (85%), 5 (83%), and 8 years post-birth (84%); 70% of all households completed all assessments over 8 years. We used data from the baseline assessment and the follow-up at eight years [1].

### Measures

#### Maternal and child sleep

We assessed sleep behaviours at eight years post-birth using the Pittsburgh Sleep Quality Index (PSQI) [26] for mothers and the Children’s Sleep Habits Questionnaire (CSHQ) for their children [27]. The PSQI focusses on seven domains of sleep (*i.e.* sleep quality, sleep latency, sleep duration, habitual sleep efficiency, sleep disturbances, use of sleep medications and daytime dysfunction) with a total of 24 items that provide a global score of subjective sleep quality. The global score ranges from 0–21, with a higher score indicating poorer subjective sleep quality. We used a cut-off score of PSQI > 5 to indicate ‘difficult sleep’, as supported by previous research [33].

The CSHQ includes 31 items focussed on several key domains of child sleep, including bedtime behaviour and sleep onset, sleep duration, anxiety around sleep, behaviour occurring during sleep and night-wakings, sleep-disordered breathing, parasomnias, and morning waking/daytime sleepiness. Multiple items of the CSHQ indicate that co-sleeping reflects sleep difficulties [24]. In many settings in South Africa, however, co-sleeping was common, and there were rarely enough beds for household members to sleep in separate beds. We eliminated these 22 items from the measure, resulting in 25 items. We asked the caregivers to recall these sleep behaviours over a ‘typical’ recent week and items were rated on a three-point scale: ‘usually’ if the sleep behaviour occurred five to seven times/week; ‘sometimes’ for two to four times/week; and ‘rarely’ for zero to one time/week. There were eight subscales: bedtime resistance, onset delay, duration, anxiety, night-waking, parasomnias, disordered breathing, and daytime sleepiness [33]. A higher CSHQ score indicates poorer subjective sleep behaviours.

#### Maternal sociodemographic characteristics

Mothers reported whether they and their children were currently living in Cape Town or the Eastern Cape, whether they had formal (*vs.* informal) housing, and whether they had access to electricity, toilets, and/or water on-site.

#### Maternal alcohol use

Alcohol use before and after recognising pregnancy was reported, as well as problematic drinking. We used the Alcohol Use Disorders Identification Test to measure alcohol use [28]. Generally, higher values indicate more problematic alcohol use; we used a cutoff score of two. Alcohol was measured at each assessment, but only scores during pregnancy and at eight years are used.

#### Maternal HIV status

HIV status was self-reported by mothers and confirmed by documentation on the child’s government-issued Road-to-Health card. We defined MLH as a mother who reported a positive HIV status at any study assessment.

#### Maternal depressive symptoms

We used the Edinburgh Postnatal Depression Scale (EPDS) [29] to measure the severity of depressive symptoms, with higher values indicating worse symptoms at each assessment. This measure has one item assessing sleep; we removed this item to examine depressive symptoms without confounding sleep reports. We examined depression only in the eight-year assessment.

#### Maternal intimate partner violence (IPV)

Mothers reported whether they had been slapped, pushed or shoved, and/or threatened with a weapon by a current partner in the past 12 months.

#### Child measures

We classified children as having low birth weight – less than 2500 grams at birth (1) or not (0). We also classified children as ever being malnourished, measured at any assessment with weight-for-age z scores (WAZ) being less than −2 standard deviations (SDs) from the World Health Organization’s (WHO) norms for weight (1) or not (0). Additionally, we classified children as being stunted, which is reflected in the height-for-age z scores (HAZ) being −2 SDs below the WHO’s norms for height (1) or not (0). We administered the Kaufman Assessment Battery for Children at eight years [30]. We used the Mental Processing Index (MPI), which measures general mental processing ability and excludes an assessment of acquired knowledge. This index has shown to be a valid, reliable, and fair measure of children’s childhood cognitive abilities in multiple settings and countries [31]. We assessed behaviour at eight years using the aggressive behaviour subscale from the Achenbach Preschool Behaviour Checklist and the Child Behaviour Checklist at three, five, and eight years [32]. Lastly, we administered the Strengths and Difficulties Questionnaire prosocial subscale [33] at eight years.

### Data analysis

**Figure 1:**
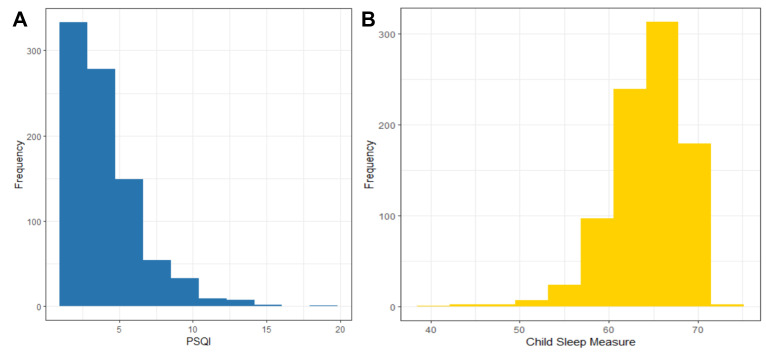
Distribution of sleep scores of mothers and children. **Panel A.** Mothers. **Panel B.** Children.

**Figure 2:**
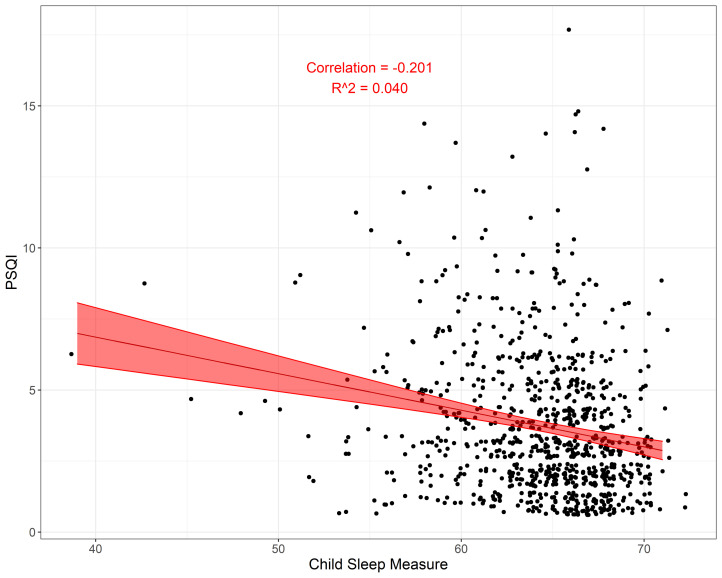
Correlation between sleep scores for mothers on the PSQI and the child sleep measure. PSQI – Pittsburgh Sleep Quality Index.

We conducted a descriptive analysis for maternal and child sample characteristics at eight years post-birth (**Figure 1**, **Figure 2**; Table S1 in the **Online Supplementary Document**). We also did a regression analysis for the mothers’ PSQI (Table S2 in the **Online Supplementary Document**) and CSHQ subscales (Table S3 in the **Online Supplementary Document**). We examined scatterplots to evaluate the relationship between mother and child sleep and fit a simple linear regression mode. Since both scores are integral values, we applied a technique known as ‘jittering’, which involves introducing a minor amount of noise to each value, to assess the concentration of the data.

We fit a linear mixed-effects model to analyse which risk factors contribute to changes in maternal sleep (Table S2 in the **Online Supplementary Document**). The linear mixed effects model uses *log* (PSQI+1) as the outcome; the *log* transformation uses the natural *log* to ensure assumptions on the residual errors are satisfied and inference is reliable. We include formal housing, water on site, electricity, living in the rural Eastern Cape, IPV, number of children, EPDS, Alcohol Use Disorders Identification Test, the natural *log* of our child sleep measure, and HIV status as fixed effects. Additionally, we controlled for any neighbourhood effect through a random effect.

Similarly, we fit a linear mixed-effects model for our child sleep measure (Table S3 in the **Online Supplementary Document**). Fixed effects included indicating the presence or absence of formal housing, water on site, electricity, living in the rural Eastern Cape, and gender. In addition, the linear mixed effects model included the Pro-Social score of the Strengths and Difficulties Questionnaire, the Aggressive Behavior sub-score of the Child Behavior Checklist, and the Kaufman MPI score, eight-year WAZ, eight-year HAZ, eight-year body mass index, history of malnourishment, history of stunting, maternal PSQI, and maternal HIV status. We included neighbourhood as a random effect.

Because of issues estimating the neighbourhood effect, we used a quasi-Bayesian linear mixed-effects model rather than a traditional frequentist model [34]. We assumed a noninformative prior distribution on the neighbourhood variance that forces our estimate to be positive. We used *R*, version 4.5.1 (R Core Team, Vienna, Austria) for all analyses. We fit our models with the blme package [35,36] and created plots with the ggplot2 [37] plotting. We used Nakagawa's method when reporting R^2^ values from linear mixed-effects models [38].

## RESULTS

In pregnancy, mothers were, on average, 26 years old (mean = 34.5). While there was a significant difference in the highest grade completed, it was less than a two-month difference, having typically stopped school at tenth grade (Table S1 in the **Online Supplementary Document**). While 54% had a partner at eight years, this was 11% more common than while pregnant. Only 19% of mothers had been employed during pregnancy, and now about half were employed (52%). Those with monthly incomes greater than ZAR 2000 went from 47% to 82% at eight years post-birth. Formal housing was up (31–56%), as was electricity (89–96%). At eight years, 65% of households had water on the premises, and half had a toilet. In addition, 29% had left Cape Town and lived in rural Eastern Cape. Among those who left, there were often multiple periods in early childhood when the mother/child went for periods to the Eastern Cape and then returned to Cape Town; of those that migrated in the first eight years, 54.6% of children migrated at least twice between Cape Town and the Eastern Cape. Most children (68.2%) lived with their mother when migrating, but grandparents and extended family raised a substantial minority. Only 8% of mothers and/or their children migrated within three years post-birth. Most migrated when children were 3–8 years of age. While 35% of mothers reported a depressed mood in pregnancy, only 6.2% did so at eight years. MLH went from 26% in pregnancy to 45% being MLH when their children were eight years of age. In pregnancy, more than a third of mothers (36%) had experienced IPV; recent IPV at eight years was 10% over the last month. Alcohol use before recognising one’s pregnancy was 25%. Almost no mothers drank alcohol in the first six months post-birth, and few had problematic alcohol use (1.1%), but this rate climbed back to pre-pregnancy levels of 25% by 8 years post-birth, with problematic use at a similar rate to post-birth.

The percentage of children with MPI scores <85 was considerably higher than expected at eight years of age (88.2%); the sample had a mean score of 73 on the MPI. The average WAZ was 0.0, and the HAZ was −0.2.

### Maternal and child sleep

Mothers’ sleep scores skew to the left, and the children’s skew to the right (**Figure 1**, Panels A and B). Among mothers, 29.5% reported sleep difficulties, with a mean sleep quality score of 3.72 (SD = 2.6). Children's sleep scores were 64.4 (SD = 4.0), with subscale scores on sleep difficulties higher than documented in HICs. Because we removed co-sleeping items, we cannot use the standardised scores from samples in HICs to specify how many children have sleep problems.

There was a weak negative correlation between maternal and child sleep (**Figure 2**). The sample correlation was −0.201 (95% CI = −0.264, −0.136), which resulted in an R^2^ value of 0.041 in the simple linear regression model. Because there may be a few outliers with CSHQ, we re-ran the analysis after removing any CSHQ values using the 1.5 × interquartile range rule. Without the outliers, we see a similar correlation of −0.199 (95% CI = −0.263, −0.134).

We conducted a regression analysis comparing these subgroups' sociodemographic and risk histories (Table S2 in the **Online Supplementary Document**), revealing that the subscales are generally higher than a sample from an HICs [39].

There was a relationship between maternal PSQI scores and living in the rural eastern cape (*P* = 0.034), experiencing IPV (*P* = 0.052), and a high score on the EPDS (*P* < 0.001) (Table S3 in the **Online Supplementary Document**). We estimated that living in the rural Eastern Cape increases *log* (PSQI+1) by an average of 0.104 (95% CI = 0.008, 0.200), indicating more sleep problems. Additionally, we estimated that IPV increases *log* (PSQI+1) by 0.107 (95% CI = −0.001, 0.215) on average, showing that IPV is related to poor sleep. We estimated an average one-unit EPDS increase *log* (PSQI+1) by 0.037 (95% CI = 0.030, 0.044). After controlling for other factors, the child sleep measure remains significantly related to maternal sleep (*P* = 0.002); for a one-unit increase in the child sleep measure, we expected a decrease of −0.013 (95% CI = −0.022, −0.005) in *log* (PSQI+1) in maternal sleep. Household features, the number of children, alcohol use, and HIV status were not significantly related to maternal sleep.

While the linear mixed effects model had some apparent and expected effects, our R^2^ value was only 0.193, indicating that our model only explains a small portion of the variance in PSQI.

Our analyses clarify a small percentage of factors contributing to sleep (R^2^ = 0.180) (Table S4 in the **Online Supplementary Document**). Also, similar to the findings for maternal sleep, we found that household features did not significantly impact child sleep. Unlike maternal sleep, child sleep did not differ between urban and rural areas. Both aggressive behaviour (*P* < 0.001) and MPI (*P* = 0.016) are significantly, inversely related to child sleep. We estimated that the child's sleep measure decreased by 0.191 points (95% CI = −0.229, −0.152) with a one-unit increase in aggressive behaviour. Similarly, we estimated that a one-unit increase in MPI decreases the child sleep measure by 0.035 points (95% CI = −0.063, −0.006). No current or lifetime growth factors were significantly related to child sleep, nor were prenatal maternal EPDS and maternal HIV status. After controlling for other factors, maternal PSQI remains significant (*P* = 0.026); a one-unit increase in PSQI is estimated to decrease the child sleep measure by 0.134 points (95% CI = −0.219, −0.013).

## DISCUSSION

We are the first to evaluate the relationship between mother and child sleep on the African continent. The rates of self-reported sleep problems are similar to those reported in studies in HICs (between 21–40% in mothers and 18–33% in children) [40,41]. Interestingly, unlike studies conducted in HICs, which have found that children’s sleep is positively associated with maternal sleep [42–44], we found a negative correlation between mother and child sleep problems. We found a significant inverse correlation between mother and child sleep problems.

It is likely that the lack of separate sleeping spaces, especially in informal and rural dwellings (65% of the sample), substantially affected mother and child sleep. Co-sleeping is far more common in South Africa than the data from HICs [45]. Given how common co-sleeping and room sharing is, and the high rates of sleep disordered breathing, parasomnias, and night awakenings reported, it is plausible that the better the mother sleeps, the worse the child sleeps due to heightened anxiety about being awake and alone at night. In Japan, toddlers and parents who share a bedroom found that their joint sleep quality is closely related to the timing of sleep – a factor we did not evaluate in our study [46]. We can speculate on the cause of this finding that differs significantly from HICs. Still, we lack solid data, either empirical or qualitative, to allow us to interpret this finding confidently.

Mothers are committed to providing a context for their children’s sound sleep, a quiet household without TV or late at night, and electric lighting, which likely requires far greater maternal vigilance. Mothers’ attention to their children’s sleep may occur at the cost of their comfort and sleep. We lack observational data on the households’ social interactions, especially at night, and, therefore, can only speculate on why mothers’ sleep is inversely related to their children’s sleep. Fortunately, we have a population cohort with relatively high retention over eight years. In addition, from having very similar living situations during pregnancy (24 matched communities which were geographically non-contiguous), migration to a deeply rural area and moving into a formal dwelling are two significant moves that occurred to one-third of the population cohort. This provides substantial variability in the sleep contexts for eight-year-old children and mothers.

Children’s sleep scores were significantly higher than comparison samples in HICs, including children with clinically disordered sleep and developmental delays [33,47]. Indeed, our sample scored one SD or more above the control sample from an HICs on six of the eight subscales of the CSHQ. These findings indicate the presence of significant sleep problems and highlight the importance of screening for sleep problems among children in LMICs. The context of small dwellings with large household sizes and few economic resources (*e.g.* having more than one bed) are likely to be contexts in which it is much harder for both mothers and children to sleep soundly all night long.

Moreover, we found that sleep problems were associated with less aggressive behaviour and lower scores on the measure of cognitive processing. The cognitive processing result is consistent with a large body of research from HICs which indicates that sleep problems are associated both cross-sectionally and longitudinally with worse cognitive outcomes. In contrast to the data from HICs [43,44,46–49], however, a positive relationship exists between aggressive behaviour and sleep quality. It may be that children who tend to be more aggressive are more aware of their needs and assert themselves to secure a more comfortable sleep environment. While we can speculate on such potential relationships, we do not have the data to support this hypothesis. Future longitudinal observations of maternal-child sleep, especially within the context of a longitudinal intervention trial to improve sleep, are needed to inform the meaning of these findings.

While children do not have a significant impact on mothers’ sleep, living in the Eastern Cape, experiencing IPV, and having a depressed mood are factors that shape mothers’ sleep. Surprisingly, none of the household characteristics – living in informal housing (or not), having water on the premises, a toilet, electricity – were related to sleep. In a deeply rural area, we anticipated that there would be fewer noises, instances of violence, and intrusions affecting sleep. Yet, sleep was more disturbed in the rural setting. Perhaps the challenges of daily living, such as getting water for 3–4 hours daily, could increase the challenges at night. Alternatively, some mothers report being more anxious about potential robberies at night, without neighbours nearby to stop thefts. The finding on depressed mood may be an artefact of this study design. There is a sleep item on the EPDS; we did not remove this item when calculating maternal depressed mood to allow standardised comparisons to other samples. This may overinflate the scores on depressed mood. However, IPV is associated with poor sleep, similar to findings from a recent meta-analysis [23]. We expected that having HIV would have been associated with sleep, similar to findings in HICs [50]; however, HIV did not affect the sleep of MLH in this South African sample. The reasons for this are unclear. When 45% of the population is HIV seropositive, HIV may possibly be less problematic for mothers, disturbing their sleep.

## CONCLUSIONS

Although maternal and child sleep in LIMCs is poorly understood, our results suggest that children growing up in crowded households and with limited resources experience higher than average sleep disturbances in middle childhood. These sleep difficulties may be associated with their cognitive functioning and behaviour. Future research in LMICs is needed to understand the relationship between the home environment and maternal and child sleep to help inform families about how to support healthy sleep patterns. Studies designed to inform the causes of this poor sleep are required to provide reliable estimates of these findings about sleep.

## Additional Material


Online Supplementary Document
