# Supplementary material for: The relationship of sleep problems between eight-year-old South African children and their mothers
Source: J Glob Health. 2025 Sep 26;15:04122. doi: 10.7189/jogh.15.04122 (PMC12471224; doi:10.7189/jogh.15.04122)
Supplement: Online Supplementary Document [file jogh-15-04122-s001.pdf]

Supplement to: Rotheram-Borus MJ, Christodoulou J, Asarnow LD, Norwood PP, Yalch M, Vogel S, Tomlinson M. The relationship of sleep problems between 8-year-old South African children and their mothers. J Glob Health. 2015;15:04122.

Table S1. The sample sociodemographic characteristics for mothers retained at 8-year follow-up broken down by maternal sleep problems (PSQI > 5)*

	No sleep problems	Sleep problems	Total
**Total number**	669	167	866
**Demographics and environmental factors**			
Age†	34.4 (5.4)	34.9 (6.0)	34.5 (5.5)
Highest level (years) of education‡	10.4 (1.8)	10.2 (2.1)	10.3 (1.9)
Married/Living with Partner‡	385 (55.1)	84 (50.6%)	469 (54.2)
Employed‡	377 (53.9)	78 (47.0)	455 (52.6)
Monthly RAND>2000‡	542 (83.4)	118 (77.1)	660 (82.2)
Formal housing‡	391 (55.9)	94 (56.6)	485 (56.1)
Water on site‡	451 (64.5)	112 (67.9)	563 (65.2)
Flush toilet‡	341 (48.8)	82 (49.4)	423 (48.9)
Electricity‡	672 (96.1)	162 (97.6)	834 (96.4)
Rural Eastern Cape‡	207 (29.6)	42 (25.3)	260 (29.4)
**Maternal risk factors**			
EPDS‡	2.1 (3.6)	6.8 (7.4)	3.0 (5.0)
EPDS > 13‡	18 (2.6)	36 (21.7)	52 (6.2)
Antenatal EPDS†	10.8 (6.8)	12.4 (6.9)	11.1 (6.8)
Antenatal EPDS > 13‡	228 (35.3)	66 (44.3)	294 (37.0)
Mothers living with HIV‡	309 (45.4)	70 (42.7)	379 (44.9)
IPV‡	54 (7.7)	34 (20.5)	88 (10.2)
AUDIT-C†	1.0 (2.3)	1.3 (2.8)	1.1 (2.4)
Number of Children†	2.7 (1.2)	2.9 (1.3)	2.7 (1.2)
**Children's risk factors**			
Low birth weight‡	n (14.5)	n (17.5)	n (15.9)
Aggressive Behavior Subscale, 8 years†	6.9 (7.0)	10.9 (8.8)	7.7 (7.5)
Kaufman MPI, 8 years*	73.6 (9.6)	73.8 (9.5)	73.6 (9.5)
WAZ, 8 years†	0.0 (1.1)	0.2 (1.3)	0.0 (1.1)
Ever WAZ <-2†	72 (10.3)	17 (10.2)	89 (10.3)
HAZ, 8 years†	-0.3 (1.0)	-0.2 (1.0)	-0.2 (1.0)
Ever HAZ <-2‡	259 (37.1)	65 (38.9)	324 (37.4)

*Some variables have missing values; percentages are adjusted accordingly.
†Values presented as mean (standard deviation).
‡Values presented as number (percentage).

Table S2. The overall score and the subscale scores of the CSHQ*

Subscale	x̄ (SD)
Child Sleep Measure	64.4 (4.0)
Bedtime Resistance	13.9 (1.4)
Onset Delay	2.7 (0.6)

Duration	5.5 (0.7)
Anxiety	10.5 (1.6)
Night Waking	7.2 (1.1)
Parasomnias	19.8 (1.6)
Disordered Breathing	8.6 (1.0)
Daytime Sleepiness	14.8 (1.8)

*The Children's Sleep Habit Questionnaire (CSHQ) had all items referring to co-sleeping removed, as these behaviors are common and accepted in the culture.

Table S3. Results for the regression of maternal sleep with potential factors associated with sleep (N = 766)*

	**Estimate (SE)**	**95% CI**	***P*-value**
Formal housing	−0.041 (0.042)	−0.122, 0.041	
Water on site	0.021 (0.040)	−0.059, 0.100	
Living in Rural Eastern Cape	0.104 (0.049)	0.008, 0.200	<0.05
Electricity on the premises	0.078 (0.094)	−0.106, 0.261	
IPV	0.107 (0.055)	−0.001, 0.215	<0.10
Number of children	0.009 (0.014)	−0.017, 0.036	
Depressive symptoms on the EPDS	0.037 (0.003)	0.030, 0.044	<0.01
AUDIT-C for problematic alcohol use	0.008 (0.007)	−0.005, 0.021	
Children's Sleep Habits Questionnaire Total Score	−0.013 (0.004)	−0.022, −0.005	<0.01
Being an MLH	−0.035 (0.034)	−0.101, 0.031	

IPV – interpersonal violence, EPDS – Edinburgh Perinatal Depression Scale, MLH – mother living with HIV

*The outcome is calculated as a Log(PSQI +1) and all analyses controlled for a neighborhood effect based on the recruitment site at birth, with a random effect. The R^2 for this model is 0.193. Also, results of the regression analyses includes children's scores on the Children's Sleep Habits Questionnaire (CSHQ).

Table S4. Results of the regression analyses for the factors associated with children's sleep on the CSHQ (N = 750)*

	**Estimate (SE)**	**95% CI**	**P-value**
Formal Housing	0.013 (0.337)	(-0.648, 0.674)	
Water on Site	-0.521 (0.312)	(-1.133, 0.091)	<0.10
Living in the Rural Eastern Cape	0.283 (0.372)	(-0.446, 1.013)	
Electricity	0.124 (0.757)	(-1.359, 1.608)	
Gender – Female	-0.313 (0.265)	(-0.832, 0.207)	
Prosocial Score	-0.078 (0.080)	(-0.236, 0.080)	
Aggressive Behavior Score	-0.191 (0.019)	(-0.229, -0.152)	<0.01
MPI	-0.035 (0.014)	(-0.063, -0.006)	<0.05
WAZ at 8 years	-0.207 (0.599)	(-1.381, 0.967)	
HAZ at 8 years	0.211 (0.393)	(-0.560, 0.982)	
BMI at 8 years	0.002 (0.194)	(-0.379, 0.383)	

Ever Malnourished from birth to 8 years	-0.304 (0.462)	(-1.210, 0.602)	
Ever Stunted from birth to 8 years	0.119 (0.316)	(-0.499, 0.738)	
Maternal Prenatal Depressive Symptoms on the Edinburg Perinatal Depression Scale	-0.031 (0.020)	(-0.070, 0.008)	
Maternal PSQI	-0.116 (0.052)	(-0.219, -0.013)	<0.05
Maternal HIV, a Mother living with HIV (MLH)	0.134 (0.269)	(-0.392, 0.663)	

*The outcome is the total score on the Children's Sleep Habits Questionnaire (CSHQ), controlling for a neighborhood effect of where the mother was initially recruited during pregnancy with a random effect. The model includes mother's total sleep score on the Pittsburgh Sleep Quality Inventory (PSQI) The R^2 for this model is 0.180.
